# Dietary Dihydromyricetin Supplementation Enhances Antioxidant Capacity and Modulates Jejunal Barrier Function, Cecal Microbiota, and Hepatic Metabolism in Mice

**DOI:** 10.3390/nu18142390

**Published:** 2026-07-22

**Authors:** Wenjiao Liang, Lishiyuan Tang, Jinghui Fan, Rui Huang, Jiaxuan Chen, Lichun Qian

**Affiliations:** 1 Key Laboratory of Animal Nutrition and Feed Science in East China, Ministry of Agriculture, College of Animal Sciences, Zhejiang University, Hangzhou 310058, China; 22417045@zju.edu.cn (W.L.); 22517082@zju.edu.cn (L.T.); 22517069@zju.edu.cn (R.H.); 22417077@zju.edu.cn (J.C.); 2 Hangzhou Academy of Agricultural Sciences, Hangzhou 310004, China; zigugu@hz.com

**Keywords:** dihydromyricetin, antioxidant status, jejunal barrier function, gut microbiota, hepatic metabolomics

## Abstract

**Background**: Dihydromyricetin (DHM) is a food-derived flavonoid enriched in vine tea and has been reported to possess antioxidant and metabolism-regulating properties. This study was designed to characterize the multi-level nutritional responses to dietary DHM supplementation, with emphasis on hepatic redox–inflammatory status, jejunal barrier-related phenotypes, cecal microbiota remodeling, hepatic metabolomic alterations, and homocysteine (Hcy) metabolism-related markers in mice. **Methods**: Forty-eight healthy mice were assigned to a basal-diet control group or diets containing 50, 100, or 200 mg/kg DHM for 4 weeks. Growth performance, serum biochemistry, antioxidant parameters, hepatic antioxidant-related expression, hepatic inflammatory cytokines, jejunal morphology and tight junction proteins, cecal 16S rRNA profiles, hepatic metabolomics, and Hcy metabolism-related markers were assessed. **Results**: Dietary DHM improved serum and hepatic antioxidant status, as reflected by increased T-AOC and GSH-Px activity and decreased MDA concentrations (*p* < 0.05). DHM also modulated the hepatic cytokine profile, with decreased TNF-α concentration (*p* < 0.05) and increased IL-10 concentration (*p* < 0.01). DHM increased hepatic Nrf2 protein abundance, HO-1 protein abundance, and Gclc mRNA expression (*p* < 0.05). DHM also improved jejunal villus architecture, as indicated by increased villus height, decreased crypt depth, and an increased villus height-to-crypt depth ratio (*p* < 0.05). Jejunal Occludin and ZO-1 protein expression were increased in the DHM-treated groups (*p* < 0.05). Cecal microbiota analysis showed increased richness and diversity indices and altered microbial community structure. Hepatic metabolomics revealed changes involving vitamin B6 metabolism, purine metabolism, the pentose phosphate pathway, and α-linolenic acid metabolism. Serum Hcy levels decreased (*p* < 0.05), accompanied by increased hepatic BHMT and MTHFR protein abundance (*p* < 0.01). **Conclusions**: Dietary DHM supplementation improved hepatic redox status and supported a less pro-inflammatory cytokine profile in mice, accompanied by enhanced jejunal barrier-related phenotypes, cecal microbiota remodeling, hepatic metabolic alterations, and Hcy metabolism-related responses. These findings provide a multi-level nutritional evaluation of DHM and suggest its potential relevance for supporting intestinal barrier integrity and hepatic metabolic homeostasis under basal physiological conditions.

## 1. Introduction

Plant-derived dietary bioactive substances have attracted increasing attention in recent years because they may help maintain metabolic homeostasis beyond meeting basic nutritional requirements. Among plant-derived bioactives, flavonoids are common in the human diet and are found in fruits, vegetables, tea, and other plant-based foods [1]. Recent evidence from reviews and prospective cohort studies suggests that both flavonoid intake level and dietary flavonoid diversity are linked to lower all-cause mortality and a reduced risk of several chronic diseases [2,3]. These findings have promoted research interest in foods that contain flavonoids and have also supported the potential application of flavonoids in nutraceutical and functional food development.

Ampelopsis grossedentata, which is commonly processed into vine tea, contains dihydromyricetin (DHM, also called ampelopsin) as one of its characteristic flavonoids [4]. Due to its polyhydroxylated structure, DHM has attracted research interest in its antioxidant properties and diverse biological functions. DHM is a promising food-derived bioactive compound, although challenges related to its stability, metabolism, and bioavailability remain [5,6]. However, most existing studies on DHM have been conducted under pathological or stress-related conditions, including diet-induced obesity or steatosis, alcohol-related liver injury, toxin exposure, and oxidative stress models [5,7,8,9]. These studies suggest that DHM may be beneficial in contexts characterized by hepatic oxidative stress, inflammatory activation, lipid metabolic disturbance, or intestinal barrier disruption. For example, alcohol exposure and overnutrition-related metabolic stress commonly involve redox imbalance, inflammatory responses, and hepatic lipid metabolic alterations, which are also closely connected with gut–liver axis regulation [9,10]. Therefore, the potential nutritional value of DHM may not be limited to disease-related protection, but may also involve the maintenance of redox balance, intestinal barrier integrity, microbial ecology, and hepatic metabolic homeostasis under basal physiological conditions. This rationale is particularly relevant to functional food and preventive nutrition research, in which dietary bioactive compounds are generally considered for health maintenance rather than disease treatment.

Redox balance, intestinal barrier integrity, gut microbial ecology, and hepatic metabolic homeostasis are all important components of nutritional homeostasis. In porcine and murine models of intestinal injury, DHM has been reported to be associated with Nrf2-related antioxidant defense [11,12]; meanwhile, flavonoids interact bidirectionally with the gut microbiota, and this interaction can affect the biotransformation and bioavailability of flavonoids [13]. In addition, Hcy metabolism is closely related to hepatic redox regulation because it connects methionine turnover, one-carbon metabolism, transsulfuration, and GSH production. Therefore, Hcy-related markers may provide useful information on hepatic metabolic status and antioxidant balance [14,15].

Accordingly, the present study was designed to characterize the multi-level nutritional responses associated with dietary DHM supplementation under basal dietary conditions in mice, with particular emphasis on hepatic redox–inflammatory status, jejunal barrier-related phenotypes, cecal microbiota composition, hepatic metabolomic profiles, and Hcy metabolism-related markers. We proposed that DHM may contribute to physiological homeostasis by enhancing redox-related responses and improving jejunal barrier-associated phenotypes, accompanied by changes in cecal microbiota, hepatic metabolism, and Hcy-related molecular markers. This multi-level evaluation provides experimental evidence for further exploring the application potential of DHM, a plant-derived dietary bioactive compound, in functional food development.

## 2. Materials and Methods

All procedures involving animals were performed under the experimental protocol approved by the Animal Ethics Committee of Zhejiang University (No. ZJU20250876).

### 2.1. Experimental Animals and Design

Twenty-eight-day-old female C57BL/6 mice were used in this study. After one week of acclimation, 48 mice with comparable initial body weights were selected for the dietary intervention. The mice were maintained in a specific pathogen-free facility with controlled housing conditions, including 21–24 °C, 50–60% relative humidity, and a 12 h light/dark cycle. Feed and water were provided ad libitum throughout the experiment. Mice were randomly divided into four groups with 12 animals in each group. This group size was used to provide sufficient biological replicates for biochemical, molecular, microbiota, and metabolomic analyses. The CON group was fed the basal diet, while the D1, D2, and D3 groups received basal diets containing 50, 100, and 200 mg/kg dihydromyricetin, respectively. The ingredient composition and nutrient levels of the basal diet are shown in Table S1.

To minimize potential confounding factors, the positions of mouse cages on the housing rack were rotated weekly to balance possible environmental variations in light exposure, temperature, and ventilation. To reduce subjective bias, the study was conducted under blind conditions. The researchers performing the experiments and those analyzing the data did not have access to the group information for each mouse.

### 2.2. Sample Collection

Following the 4-week dietary intervention, feed was withdrawn from the mice for 12 h, while water remained available ad libitum. Blood samples were then obtained. Mice were euthanized by intraperitoneal administration of sodium pentobarbital at 80 mg/kg according to the approved animal ethics protocol. Liver and intestinal tissues were rapidly excised after euthanasia, and liver weight was recorded immediately. Cecal contents were collected with care, placed into cryogenic tubes, and stored at −80 °C until further analysis. For histological examination, selected liver and jejunal tissues were preserved in paraformaldehyde. The other liver and intestinal samples were stored at −80 °C for later experimental analyses.

### 2.3. Measurement of Growth Performance and Serum Biochemical Parameters

Body weight was measured at five scheduled time points during the trial: days 0, 7, 14, 21, and 28. These records were used to calculate body weight gain (BWG). BWG was determined as the difference between final and initial body weight.

Following blood collection, the samples were maintained at room temperature for 2 h before serum separation. They were centrifuged at 3000 rpm for 15 min at 4 °C, and the serum was collected for biochemical analysis. Commercial kits from Nanjing Jiancheng Bioengineering Institute (Nanjing, China) were used to determine serum indices associated with hepatic function, renal function, and lipid metabolism. The measured indices included alanine aminotransferase (ALT), aspartate aminotransferase (AST), alkaline phosphatase (ALP), total bilirubin (TBil), creatinine (Crea), urea, triglycerides (TG), total cholesterol (Tchol), low-density lipoprotein cholesterol (LDL-C), and high-density lipoprotein cholesterol (HDL-C).

### 2.4. Determination of Serum Hcy, Antioxidant Indices, and Hepatic Inflammatory Cytokines

Liver tissue samples weighing 0.1 g were homogenized on ice with physiological saline at a 1:9 ratio. After centrifugation at 3000 rpm for 10 min at 4 °C, the supernatants were collected. Part of the supernatant was analyzed immediately, and the remaining sample was stored at −80 °C for subsequent assays.

Serum homocysteine (Hcy) was measured together with antioxidant-related indices in serum and liver homogenate supernatants. These indices included malondialdehyde (MDA), superoxide dismutase (SOD), glutathione peroxidase (GSH-Px), catalase (CAT), and total antioxidant capacity (T-AOC). In addition, hepatic inflammatory cytokines, including tumor necrosis factor-α (TNF-α), interleukin-1β (IL-1β), interleukin-6 (IL-6), and interleukin-10 (IL-10), were measured in liver homogenate supernatants using commercial ELISA kits. Commercial assay kits from Nanjing Jiancheng Bioengineering Institute were used according to the manufacturer’s instructions.

The Hcy metabolism-related measurements consisted of serum homocysteine (Hcy) concentration and hepatic expression of betaine-homocysteine S-methyltransferase (BHMT) and methylenetetrahydrofolate reductase (MTHFR) at both mRNA and protein levels. Serum Hcy was measured using a commercial assay kit, whereas hepatic BHMT and MTHFR expression were analyzed by quantitative real-time polymerase chain reaction (qRT-PCR) and Western blotting.

### 2.5. Hematoxylin and Eosin Staining

Jejunal and liver tissues were immersed in 4% paraformaldehyde for 24 h before paraffin embedding and section preparation. The prepared sections were then subjected to hematoxylin and eosin (H&E) staining using routine protocols. Morphological indices of the jejunum, including villus height, crypt depth, and the villus height-to-crypt depth ratio, were measured with ImageJ 1.53t.

### 2.6. Quantitative Real-Time Polymerase Chain Reaction

Jejunal and liver tissue samples were processed for total RNA extraction using TRIzol reagent, following the manufacturer’s protocol. The isolated RNA was reverse-transcribed into complementary DNA (cDNA) with a reverse transcription kit. The obtained cDNA was then used as the template for gene amplification. Target gene expression was analyzed by quantitative real-time polymerase chain reaction (qRT-PCR) using the SYBR Premix Ex Taq™ kit. Relative mRNA expression was calculated with the 2^−ΔΔCt^ method. GAPDH served as the internal reference gene for normalization. The primer sequences are shown in Table 1.

### 2.7. Western Blotting

Liver and jejunal tissue samples, each weighing 100 mg, were homogenized with 900 μL of lysis buffer containing protease inhibitor at a dilution of 1:100 (*v*/*v*) for total protein extraction. After centrifugation at 12,000 rpm for 20 min at 4 °C, the supernatants from total protein lysates were obtained for subsequent assays. Protein concentration was determined using a bicinchoninic acid (BCA) assay kit. The samples were then mixed with loading buffer at 4:1 (*v*/*v*) and denatured by heating at 100 °C for 10 min. Equal protein samples were subjected to sodium dodecyl sulfate-polyacrylamide gel electrophoresis (SDS-PAGE) and then electrotransferred onto membranes. To reduce nonspecific binding, the membranes were incubated with 5% skim milk at room temperature for 2 h, followed by three rinses in TBST buffer.

Primary antibodies targeting Nrf2, HO-1, ZO-1, Occludin, MTHFR, and BHMT were applied to the membranes overnight at 4 °C, with each antibody diluted to 1:1000. β-Actin (1:1000) was selected as the loading control for Nrf2, HO-1, ZO-1, and Occludin, while GAPDH (1:1000) was used for normalization of MTHFR and BHMT. Following incubation with the primary antibodies, the membranes were washed three times in TBST buffer. They were then incubated with horseradish peroxidase (HRP)-conjugated goat anti-rabbit secondary antibody at a dilution of 1:10,000 for 2 h at room temperature. Protein signals were developed using enhanced chemiluminescence (ECL) reagent. The band intensities were measured with ImageJ software, and the abundance of each target protein was calculated after normalization to the corresponding internal control.

### 2.8. Immunohistochemistry Staining

Jejunal paraffin sections were first treated with xylene to remove paraffin and then passed through graded ethanol solutions for rehydration. For antigen unmasking, tissue sections were heated in 0.01 M citrate buffer (pH 6.0) at 95 °C for 20 min. Endogenous peroxidase was then inactivated by treating the sections with 3% hydrogen peroxide (H_2_O_2_) prepared in deionized water for 30 min at room temperature. After that, 5% normal serum was applied for 1 h to reduce nonspecific staining. Anti-ZO-1 antibody (1:100) or anti-Occludin antibody (1:100) was applied to the sections, followed by overnight incubation at 4 °C. Staining was visualized with 3,3′-diaminobenzidine (DAB), and the development time was controlled according to the staining intensity. At the final step, the sections were stained with hematoxylin for counterstaining, dehydrated through an ethanol gradient, cleared in xylene, and sealed with neutral resin. The prepared slides were then observed and imaged using a light microscope. The prepared sections were digitized with a digital pathology slide scanner (Servicebio, Wuhan, China) to obtain whole-slide digital images. The staining results were subsequently examined and recorded using SlideViewer 2.7, and representative fields were selected at the same magnification for image presentation.

### 2.9. Cecal Microbiota 16S rRNA Sequencing

Fresh cecal contents were collected into sterile centrifuge tubes, quickly frozen with liquid nitrogen, and maintained at −80 °C before sequencing analysis. Microbial genomic DNA was obtained from these samples with the Omega Bio-tek DNA extraction kit (Norcross, GA, USA), following the supplier’s protocol. The quality of extracted DNA was evaluated using both gel-based and spectrophotometric methods. DNA integrity and fragment size distribution were assessed on a 1.0% (*w*/*v*) agarose gel run at 120 V for 30 min. DNA quantity and purity were determined with a NanoDrop ND-2000 instrument (Thermo Scientific Inc., Waltham, MA, USA). Samples showing A260/A280 ratios within the range of 1.8–2.0 were selected for downstream amplification.

The V3–V4 region of bacterial 16S rRNA was targeted for amplification using the primer pair 341F (5′-CCTAYGGGRBGCASCAG-3′) and 806R (5′-GGACTACHVGGGTWTCTAAT-3′). The polymerase chain reaction (PCR) system had a final volume of 25 μL and was prepared with 12.5 μL of 2× Taq Master Mix, 0.5 μL forward primer, 0.5 μL reverse primer, and 50 ng template DNA. Both primers were used at a concentration of 10 μM. Thermal cycling was performed with an initial denaturation step at 95 °C for 3 min, followed by 30 cycles consisting of 95 °C for 30 s, 55 °C for 30 s, and 72 °C for 45 s. The reaction ended with an extension step at 72 °C for 10 min. The amplified products were examined on a 2% agarose gel at 120 V for 30 min. Amplicons meeting the quality requirements were purified with the AxyPrep DNA Gel Extraction Kit and then measured on a Qubit 3.0 fluorometer (Promega, Madison, WI, USA). Qualified libraries were subjected to sequencing with the Illumina MiSeq system (Illumina, San Diego, CA, USA), using a 2 × 300 bp paired-end strategy.

Quality control of the raw paired-end reads was performed using fastp software (version 0.23.4) [16]. Low-quality bases at the ends of reads were removed when their quality scores were below 20. A sliding window of 50 bp was further applied to remove low-quality downstream segments when the average Phred value within that region fell below Q20. Reads were discarded if their length was less than 50 bp after filtering or if they contained more than five ambiguous N bases. The high-quality paired-end reads were assembled according to their overlap regions with FLASH (version 1.2.11) [17]. The overlap length for merging was required to be at least 10 bp, and the mismatch proportion within the overlapped segment was limited to 0.2 or less. After assembly, sequences were assigned to samples and corrected for orientation according to the barcode and primer information. No barcode mismatches were allowed, and no more than two primer mismatches were permitted.

Cleaned and merged reads were denoised through the QIIME2 workflow under default settings, resulting in amplicon sequence variants (ASVs). The representative ASV sequences were subsequently classified taxonomically against the SILVA 138.2 16S rRNA bacterial database. This step used the classify-sklearn algorithm in QIIME2 with a Naive Bayes classifier, and the confidence threshold was set at 70% [18].

Microbial alpha and beta diversity were analyzed with Mothur software (version 1.30.2), and related figures were generated in R software (version 3.3.1). Alpha diversity was used to describe microbial richness and diversity within each sample. Beta diversity was assessed using Bray–Curtis distance and displayed by principal coordinates analysis (PcoA) to compare microbial community structures among groups. Shared and group-specific taxa were examined with the online Venn tool (version 2.4.3). Microbial community patterns were further shown using bar plots and heatmaps. Differences among groups were tested in R using the Kruskal–Wallis test for multiple comparisons based on the mean abundance of differential features. Linear discriminant analysis Effect Size (LefSe) was used to screen taxa that contributed to group separation, with a linear discriminant analysis (LDA) score threshold greater than 2.5.

### 2.10. Non-Targeted Profiling of Metabolites in Liver

Liver tissue samples of approximately 50 mg were placed in 2 mL tubes, and one 6 mm bead was added to each tube for grinding. Each sample received 400 μL of chilled extraction solvent, which consisted of methanol and water at 4:1 (*v*/*v*). The solvent also contained internal standards, with L-2-chlorophenylalanine included at 0.02 mg/mL. The samples were ground for 6 min in a low-temperature tissue grinder operated at −10 °C and 50 Hz. Next, the homogenates were subjected to ultrasonic treatment under cold conditions at 5 °C and 40 kHz for 30 min. The extracts were then kept at −20 °C for 30 min before centrifugation at 13,000× *g* for 15 min at 4 °C. The clear supernatants were transferred to insert-equipped vials for liquid chromatography–mass spectrometry (LC-MS) analysis. For quality control (QC), 20 μL aliquots taken from all samples were combined and mixed to generate pooled QC samples. These QC samples were processed in parallel with the study samples and analyzed under the same LC-MS conditions.

Chromatographic separation was carried out on a UHPLC-Q Exactive HF system (Thermo Fisher Scientific, Waltham, MA, USA). Separation was performed using an ACQUITY UPLC HSS T3 column (100 mm × 2.1 mm i.d., 1.8 μm; Waters, Milford, MA, USA), and the column temperature was maintained at 40 °C. Mobile phase A was prepared from water and acetonitrile at 95:5 (*v*/*v*) with 0.1% formic acid. Mobile phase B contained acetonitrile, isopropanol, and water at 47.5:47.5:5 (*v*/*v*/*v*), also with 0.1% formic acid. For each run, 3 μL of sample solution was injected. Mass spectrometric analysis was performed with an electrospray ionization source, and spectra were acquired in both positive and negative ionization modes. The acquisition range was set from 70 to 1050 *m*/*z*. Spray voltage was 3500 V in positive mode and −3200 V in negative mode. The sheath gas and auxiliary gas flow rates were set at 50 arb and 13 arb, respectively. The ion source temperature was maintained at 425 °C. Collision energies of 20, 40, and 60 V were applied. The mass resolution was set to 60,000 for MS1 and 15,000 for MS2.

Following liquid chromatography–mass spectrometry (LC-MS) acquisition, the raw datasets were imported into Progenesis QI software (version 3.0; Waters Corporation, Milford, MA, USA) for preprocessing. The workflow included baseline filtering, peak detection, peak area integration, retention time correction, and peak alignment. After preprocessing, a data matrix was generated to record retention time, *m*/*z* values, and peak intensities. Metabolite annotation was performed by matching mass spectrometry (MS) and tandem mass spectrometry (MS/MS) data with the Human Metabolome Database (HMDB), METLIN, NIST, MassBank, and the Majorbio in-house database.

The processed feature table was submitted to the Majorbio Cloud Platform for downstream statistical processing. Features with a high proportion of missing entries were first discarded according to the 20% rule. Any missing entries left after this filtering step were filled with the smallest value observed in the original data matrix. Peak intensities were then corrected by sum normalization to minimize variation caused by sample preparation and instrumental fluctuation. Features showing a relative standard deviation (RSD) greater than 30% across quality control (QC) samples were excluded. After log10 transformation, the resulting data matrix was used for multivariate analysis. Principal component analysis (PCA) and partial least squares discriminant analysis (PLS-DA) were carried out with the ropls package in R (version 1.6.2). Seven-fold cross-validation was applied to evaluate model robustness. Differential metabolites were identified using variable importance in projection (VIP) values from the PLS-DA model together with Student’s *t*-test. Metabolites satisfying VIP > 1 and *p* < 0.05 were defined as significantly different metabolites. These metabolites were further annotated against the Kyoto Encyclopedia of Genes and Genomes (KEGG) database. For pathway enrichment, Fisher’s exact test was performed with the scipy.stats package to detect metabolic pathways linked to DHM treatment.

### 2.11. Data Analysis

All statistical analyses were conducted with IBM SPSS Statistics 23. Differences among groups were tested using one-way analysis of variance (ANOVA). When significant effects were detected, Tukey’s honestly significant difference (HSD) test was applied for post hoc multiple comparisons. Results are reported as mean ± standard error of the mean (SEM). A value of *p* < 0.05 was considered statistically significant. GraphPad Prism 10.1 was used for figure preparation and statistical result checking.

## 3. Results

### 3.1. Growth Performance of Mice

**Figure 1:**
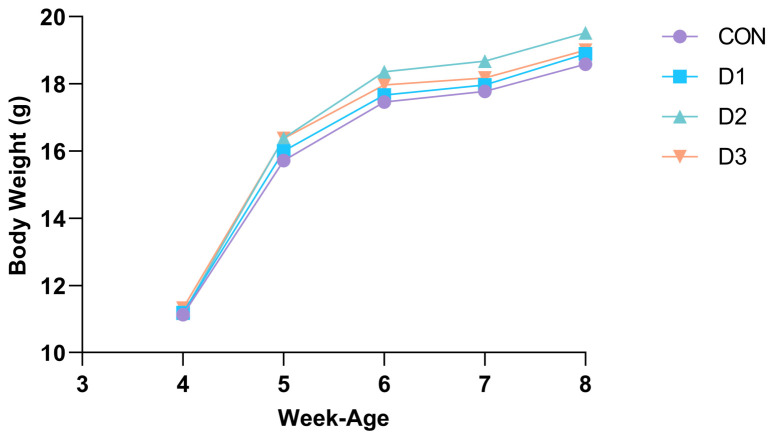
Body weight changes in mice receiving dietary DHM.

To assess the effect of dietary DHM supplementation on growth performance in mice, body weight gain (BWG) was calculated during the 28-day trial. Before the dietary intervention, the mean initial body weight of all mice was 11.21 ± 0.10 g, with no significant difference among the CON, D1, D2, and D3 groups (*p* = 0.941). Dietary dihydromyricetin increased BWG from day 0 to day 21, with a significant increase in the D2 group (*p* < 0.05). These results are presented in Figure 1 and Table 2. The liver index was not significantly different among groups (*p* > 0.05; Table S2).

### 3.2. Serum Biochemical Parameters

**Figure 2:**
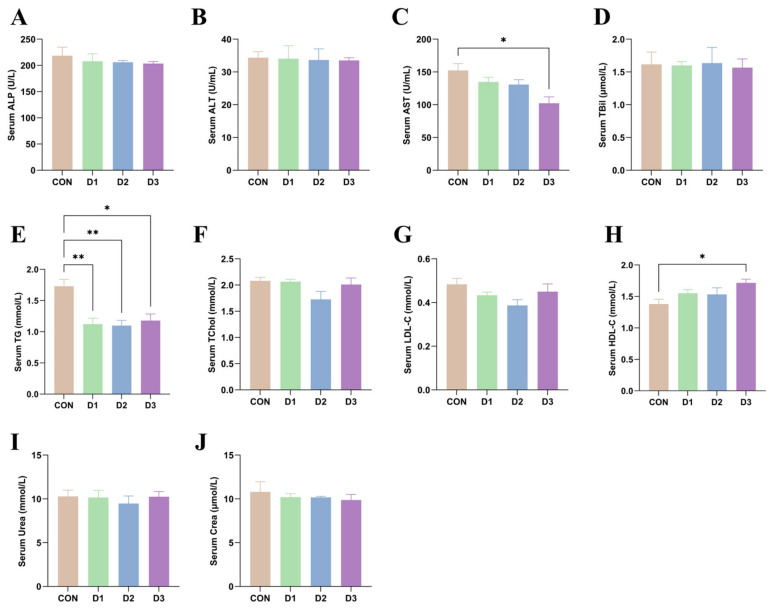
Serum biochemical responses of mice to dietary DHM supplementation. (**A**) Alkaline phosphatase (ALP); (**B**) alanine aminotransferase (ALT); (**C**) serum aspartate aminotransferase (AST); (**D**) total bilirubin (TBil); (**E**) triglycerides (TG); (**F**) total cholesterol (TChol); (**G**) low-density lipoprotein cholesterol (LDL-C); (**H**) high-density lipoprotein cholesterol (HDL-C); (**I**) urea (Urea); and (**J**) creatinine (Crea). Data are expressed as the mean ± standard error of the mean (SEM). Significant differences are indicated by “*”, “**” (* *p* < 0.05, ** *p* < 0.01); CON, basal diet; D1, D2, and D3 refer to mice receiving the basal diet plus DHM at 50, 100, and 200 mg/kg diet, respectively.

Serum biochemical parameters were analyzed to assess whether dietary DHM affected the basal physiological status of mice. For liver function-related indices, relative to mice fed the basal diet, DHM-supplemented mice showed lower serum AST activity, whereas ALT, ALP, and Tbil remained comparable across treatments (*p* > 0.05). These results are presented in Figure 2A–D. For lipid metabolism-related indices, dietary DHM led to a reduction in TG concentration (*p* < 0.01) and an elevation in HDL-C concentration (*p* < 0.05). Tchol remained largely unchanged, as did LDL-C (*p* > 0.05). These data are shown in Figure 2E–H. In addition, Crea and Urea were similar across all groups (*p* > 0.05; Figure 2I,J).

### 3.3. Liver Histological Morphology and Hepatic Inflammatory Cytokine Concentrations

**Figure 3:**
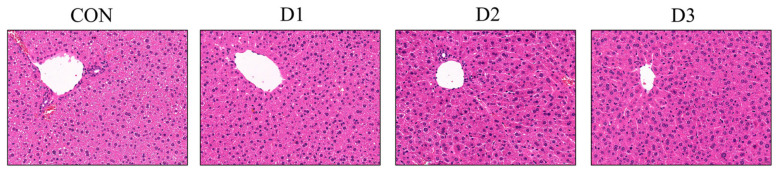
Effects of dietary DHM supplementation on hepatic histological morphology in mice. Representative H&E-stained images of mouse liver tissue are shown (400×). CON, basal diet; D1, D2, and D3 refer to mice receiving the basal diet plus DHM at 50, 100, and 200 mg/kg diet, respectively.

H&E staining was used to examine liver morphology (Figure 3). The overall hepatic architecture was well preserved across the CON, D1, D2, and D3 groups. Small cytoplasmic vacuole-like areas appeared more noticeable in the CON group than in the DHM-supplemented groups; however, no evident macrovesicular steatosis, extensive inflammatory cell infiltration, necrosis, fibrous septa, or fibrosis-related architectural changes were observed.

**Figure 4:**
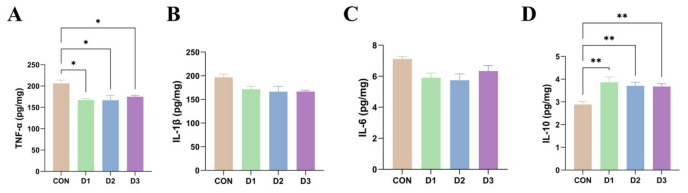
Effects of dietary DHM supplementation on hepatic inflammatory cytokine concentrations in mice. (**A**) Hepatic TNF-α; (**B**) hepatic IL-1β; (**C**) hepatic IL-6; (**D**) hepatic IL-10. Data are presented as the mean ± standard error of the mean (SEM). Significant differences are indicated by “*”, “**” (* *p* < 0.05, ** *p* < 0.01); CON, basal diet; D1, D2, and D3 refer to mice receiving the basal diet plus DHM at 50, 100, and 200 mg/kg diet, respectively.

To further evaluate hepatic inflammatory status, the concentrations of TNF-α, IL-1β, IL-6, and IL-10 in liver tissues were determined by ELISA (Figure 4). Compared with the CON group, dietary DHM supplementation significantly decreased hepatic TNF-α concentration (*p* < 0.05) and significantly increased IL-10 concentration (*p* < 0.01). Hepatic IL-1β and IL-6 concentrations showed decreasing trends in the DHM-supplemented groups, although these differences did not reach statistical significance.

### 3.4. Serum and Hepatic Antioxidant Parameters

**Figure 5:**
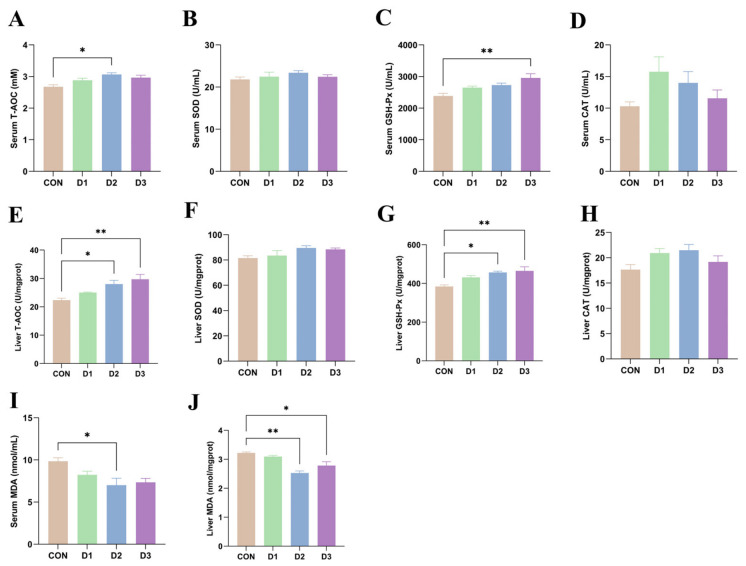
Effects of dietary DHM supplementation on serum and hepatic antioxidant capacity in mice. Serum and hepatic antioxidant capacity-related indices were measured using samples collected on day 28, at the end of the 4-week dietary intervention. (**A**) Serum total antioxidant capacity (T-AOC); (**B**) serum superoxide dismutase (SOD); (**C**) serum glutathione peroxidase (GSH-Px); (**D**) serum catalase (CAT); (**E**) hepatic total antioxidant capacity (T-AOC); (**F**) hepatic superoxide dismutase (SOD); (**G**) hepatic glutathione peroxidase (GSH-Px); (**H**) hepatic catalase (CAT); (**I**) serum malondialdehyde (MDA); and (**J**) hepatic malondialdehyde (MDA). Data are presented as the mean ± standard error of the mean (SEM). Significant differences are indicated by “*”, “**” (* *p* < 0.05, ** *p* < 0.01); CON, basal diet; D1, D2, and D3 refer to mice receiving the basal diet plus DHM at 50, 100, and 200 mg/kg diet, respectively.

Serum and hepatic antioxidant parameters were measured to assess the antioxidant response of mice to dietary DHM supplementation (Figure 5). In serum (Figure 5A–D,I), dietary DHM supplementation significantly increased GSH-Px (*p* < 0.05) and T-AOC activity (*p* < 0.01), while significantly decreasing malondialdehyde (MDA) levels (*p* < 0.05). Serum SOD and CAT activities tended to increase, but this trend was not significant (*p* > 0.05). In the liver (Figure 5E–H,J), DHM improved antioxidant status, as shown by higher T-AOC and GSH-Px activity and lower MDA levels (all *p* < 0.01). Hepatic SOD and CAT activities showed a modest upward trend, but these changes were not significant (*p* > 0.05).

### 3.5. Hepatic Antioxidant-Related Gene and Protein Expression

**Figure 6:**
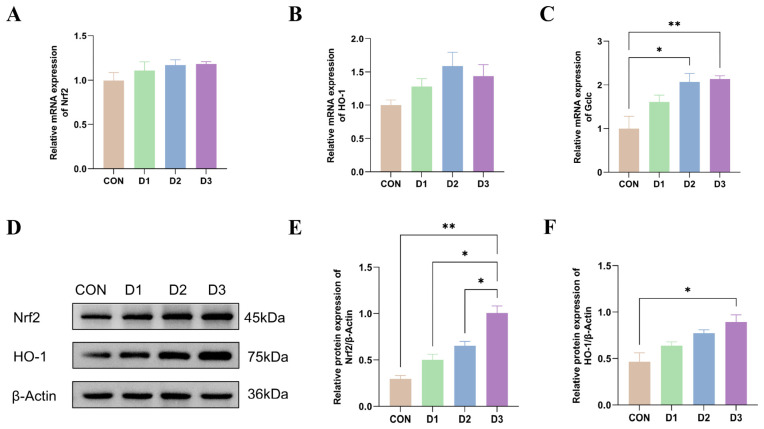
Hepatic antioxidant-related molecular responses in mice receiving dietary DHM. (**A**–**C**) Hepatic mRNA levels of Nrf2, HO-1, and Gclc; (**D**) representative Western blot bands for Nrf2 and HO-1 in liver tissue; (**E**,**F**) densitometric quantification of Nrf2 and HO-1 protein abundance. Data are presented as the mean ± standard error of the mean (SEM). Significant differences are indicated by “*”, “**” (* *p* < 0.05, ** *p* < 0.01); CON, basal diet; D1, D2, and D3 refer to mice receiving the basal diet plus DHM at 50, 100, and 200 mg/kg diet, respectively.

The qRT-PCR analysis revealed a numerical increase in hepatic Nrf2 and HO-1 mRNA expression after DHM supplementation, but the changes were not significant (*p* > 0.05; Figure 6A,B). In contrast, hepatic Gclc mRNA expression was significantly elevated in response to dietary DHM (*p* < 0.05; Figure 6C).

Western blotting further revealed an increase in hepatic Nrf2 protein abundance after dietary DHM supplementation (Figure 6D–F). Nrf2 protein abundance was elevated in D1 and D2 relative to CON (*p* < 0.05), and a stronger increase was found in D3 (*p* < 0.01; Figure 6E). For HO-1, protein abundance also increased numerically in the DHM-supplemented groups, reaching significance in D3 (*p* < 0.05; Figure 6F).

### 3.6. Jejunal Morphology and Tight Junction Protein Expression

**Figure 7:**
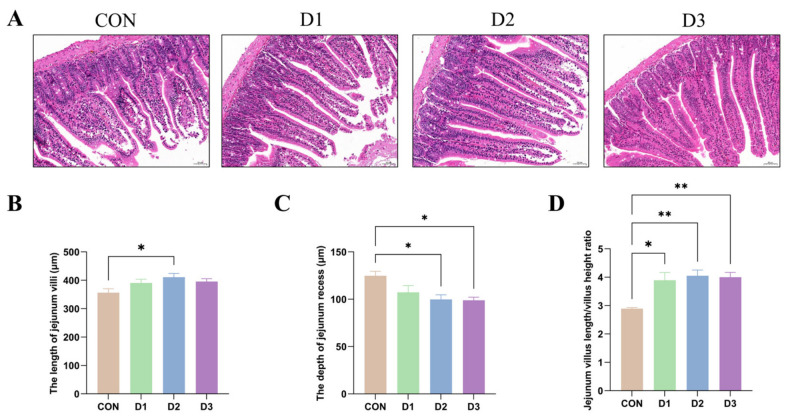
Effects of dihydromyricetin on jejunal morphology in mice. (**A**) Representative H&E-stained images of mouse jejunal tissue (200×; Scale bar = 100 μm); (**B**) jejunal villus height; (**C**) jejunal crypt depth; (**D**) villus height-to-crypt depth ratio. Data are presented as the mean ± standard error of the mean (SEM). Significant differences are indicated by “*”, “**” (* *p* < 0.05, ** *p* < 0.01); CON, basal diet; D1, D2, and D3 refer to mice receiving the basal diet plus DHM at 50, 100, and 200 mg/kg diet, respectively.

H&E staining was performed to assess jejunal morphology after dietary DHM supplementation (Figure 7A). Compared with mice fed the basal diet, DHM-supplemented mice showed increased villus height, with a significant effect in the D2 group (*p* < 0.05; Figure 7B). Crypt depth was lower after DHM supplementation, and significant reductions were detected in the D2 and D3 groups (*p* < 0.05; Figure 7C). In addition, DHM supplementation significantly increased the villus height-to-crypt depth ratio (*p* < 0.01; Figure 7D).

**Figure 8:**
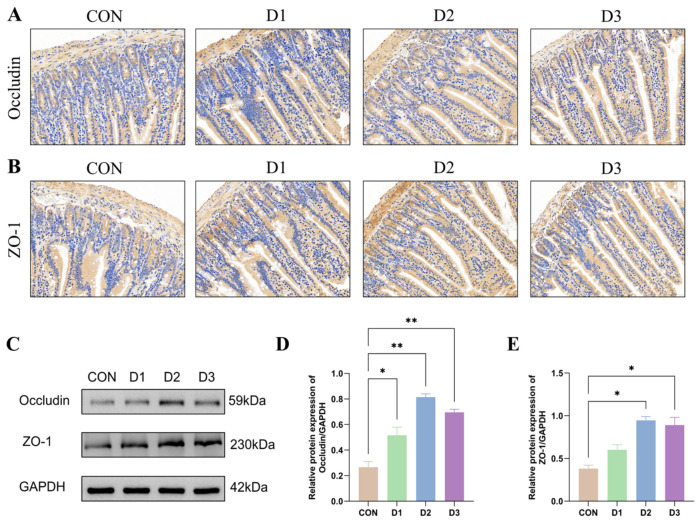
Tight junction protein expression of Occludin and ZO-1 in mouse jejunum after dietary dihydromyricetin supplementation. (**A**) Representative immunohistochemical staining images of jejunal Occludin (400×); (**B**) representative immunohistochemical staining images of jejunal ZO-1 (400×); (**C**) representative Western blot images of Occludin and ZO-1 proteins; (**D**) quantitative analysis of relative Occludin protein expression; (**E**) quantitative analysis of relative ZO-1 protein expression. Data are presented as the mean ± standard error of the mean (SEM). Significant differences are indicated by “*”, “**” (* *p* < 0.05, ** *p* < 0.01); CON, basal diet; D1, D2, and D3 refer to mice receiving the basal diet plus DHM at 50, 100, and 200 mg/kg diet, respectively.

Jejunal tight junction proteins were evaluated using immunohistochemistry and Western blotting (Figure 8). Immunohistochemistry revealed stronger Occludin and ZO-1 staining in the jejunum of DHM-supplemented mice than in mice fed the basal diet (Figure 8A,B). Consistently, Western blotting confirmed that dietary DHM significantly elevated jejunal ZO-1 and Occludin protein abundance (Figure 8C–E). In addition, dietary DHM supplementation reduced serum LPS concentration compared with the CON group (*p* < 0.05; Figure S1).

### 3.7. Cecal Microbiota Diversity and Community Structure

**Figure 9:**
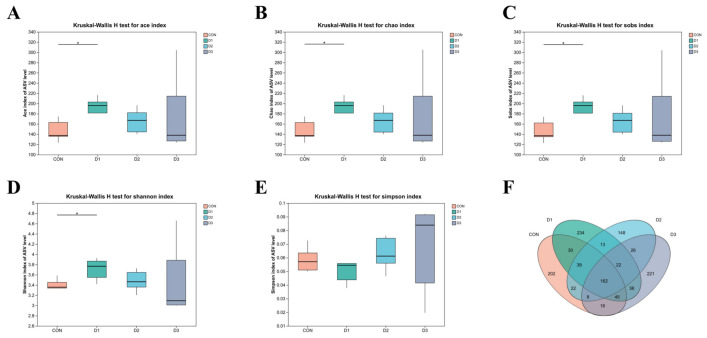

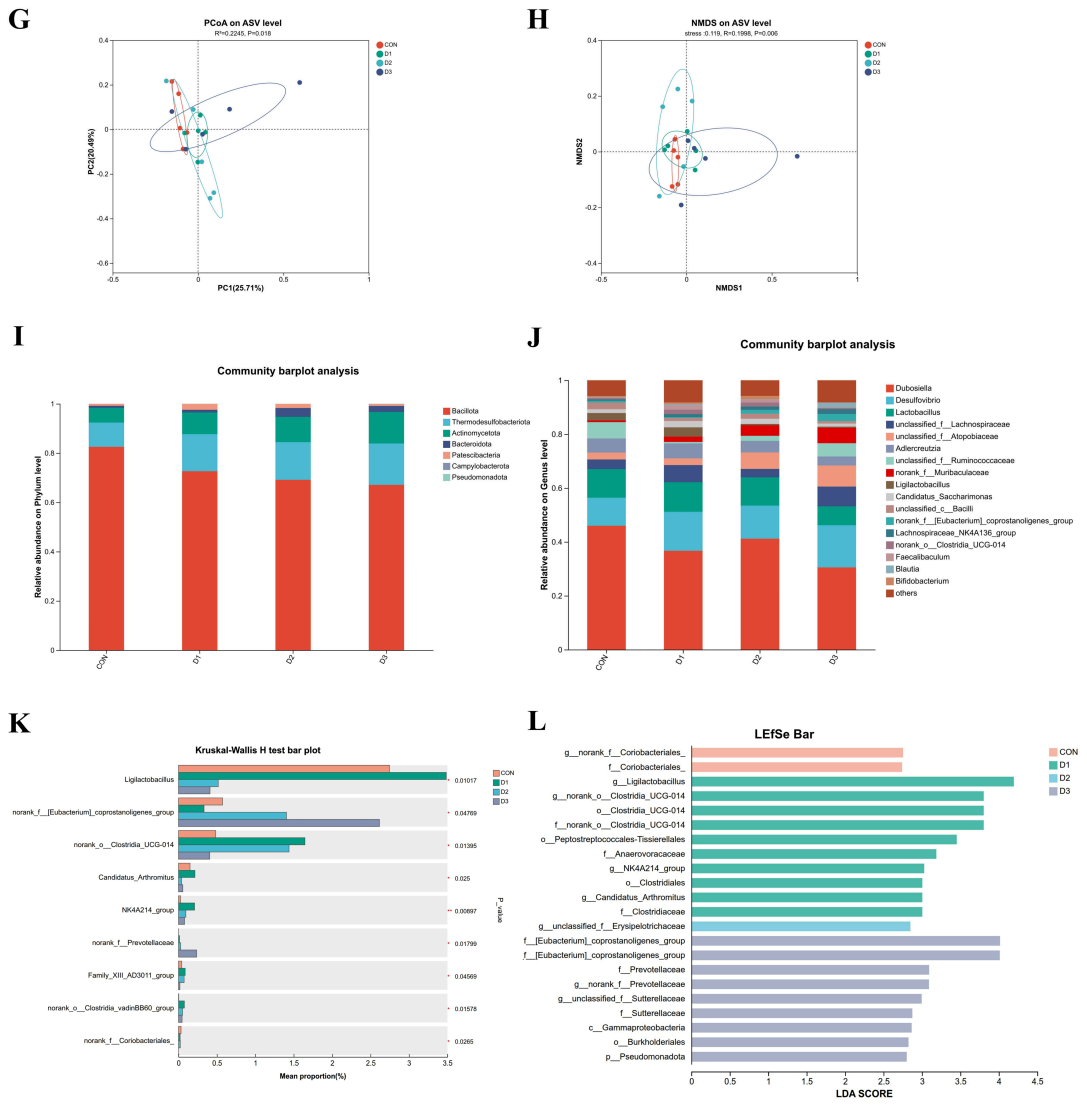
Cecal microbiota profiles in mice receiving dietary DHM. (**A**–**E**) Alpha diversity analysis based on ACE, Chao1, Sobs, Shannon, and Simpson indices; (**F**) Venn diagram showing shared and group-specific amplicon sequence variants (ASVs); (**G**,**H**) Bray–Curtis-based beta diversity shown by principal coordinates analysis (PCoA) and non-metric multidimensional scaling (NMDS); (**I**,**J**) taxonomic profiles at the phylum and genus levels; (**K**) dominant differential genera based on mean relative abundance; (**L**) linear discriminant analysis effect size (LEfSe) histogram. Significant differences are indicated by “*”, “**” (* *p* < 0.05, ** *p* < 0.01); CON, basal diet; D1, D2, and D3 refer to mice receiving the basal diet plus DHM at 50, 100, and 200 mg/kg diet, respectively.

DHM supplementation changed the alpha diversity profile of the cecal microbiota. ACE, Sobs, and Chao values were increased in the DHM-supplemented groups relative to CON, indicating higher microbial richness (Figure 9A–C). The Shannon index was also elevated in DHM-supplemented mice (*p* < 0.05; Figure 9D), whereas the Simpson index showed no clear group-dependent change (Figure 9E). The distribution of shared and unique amplicon sequence variants (ASVs) was further summarized using a Venn diagram. In total, 1256 ASVs were detected among all samples, including 162 ASVs shared by the four groups (Figure 9F). The numbers of ASVs specific to CON, D1, D2, and D3 were 202, 234, 148, and 221, respectively.

Bray–Curtis-based beta diversity analysis was used to evaluate differences in cecal microbiota structure (Figure 9G,H). The NMDS plot showed a clear separation of CON, D1, D2, and D3 samples (Figure 9H). This separation was supported by statistical analysis, which showed marked differences among the four groups (*p* < 0.01). PcoA analysis produced a consistent pattern and confirmed the group-dependent shift in cecal microbiota composition (*p* < 0.01; Figure 9G).

Phylum-level taxonomic profiling showed that Bacillota, Thermodesulfobacteriota, Actinomycetota, and Bacteroidota were the dominant phyla (Figure 9I). Compared with the CON group, dietary DHM supplementation altered the relative abundance of dominant phyla, with a decreasing trend in Bacillota and an increasing trend in Bacteroidota, resulting in a lower Bacillota/Bacteroidota ratio. Genus-level taxonomic profiling showed that the dominant genera mainly included Dubosiella, Desulfovibrio, Lactobacillus, unclassified_f__Lachnospiraceae, unclassified_f__Atopobiaceae, Adlercreutzia, unclassified_f__Ruminococcaceae, norank_f__Muribaculaceae, Ligilactobacillus, and Candidatus_Saccharimonas (Figure 9J). To examine changes in the abundance of differential taxa among groups, the mean relative abundance of these genera was compared among the CON, D1, D2, and D3 groups (Figure 9K). Compared with the CON group, DHM treatment significantly increased the relative abundance of Ligilactobacillus, norank_f__[Eubacterium]_coprostanoligenes_group, norank_o__Clostridia_UCG-014, NK4A214_group, norank_f__Prevotellaceae, Family_XIII_AD3011_group, and norank_o__Clostridia_vadinBB60_group, while decreasing the relative abundance of norank_f__Coriobacteriales.

LefSe analysis was performed to identify cecal taxa with differential relative abundance across dietary treatments (Figure 9L). The main discriminative taxa included Pseudomonadota, Gammaproteobacteria, Burkholderiales, Clostridiales, Peptostreptococcales-Tissierellales, Prevotellaceae, Sutterellaceae, Anaerovoracaceae, Clostridiaceae, Erysipelotrichaceae, Ligilactobacillus, Candidatus_Arthromitus, and [Eubacterium]_coprostanoligenes_group. The CON group was mainly enriched in Coriobacteriales, whereas the D1 group was enriched in Ligilactobacillus, Clostridia_UCG-014, Anaerovoracaceae, and Clostridiaceae. The D2 group was characterized by enrichment of unclassified Erysipelotrichaceae, while the D3 group was enriched in Prevotellaceae, Sutterellaceae, Gammaproteobacteria, Burkholderiales, and Pseudomonadota.

### 3.8. Hepatic Untargeted Metabolomic Analysis

#### 3.8.1. Comparative Analysis of Samples

**Figure 10:**
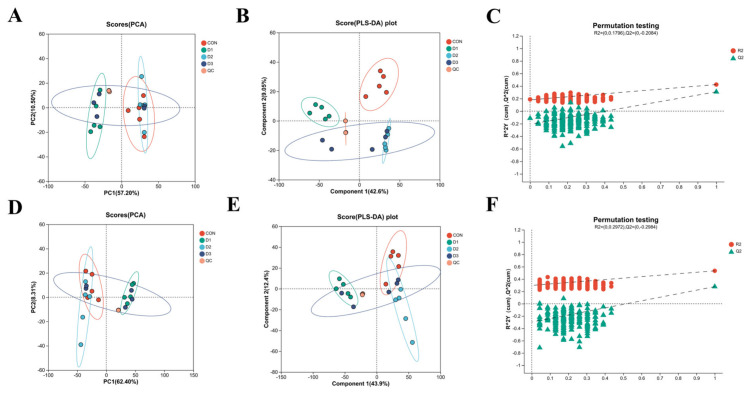

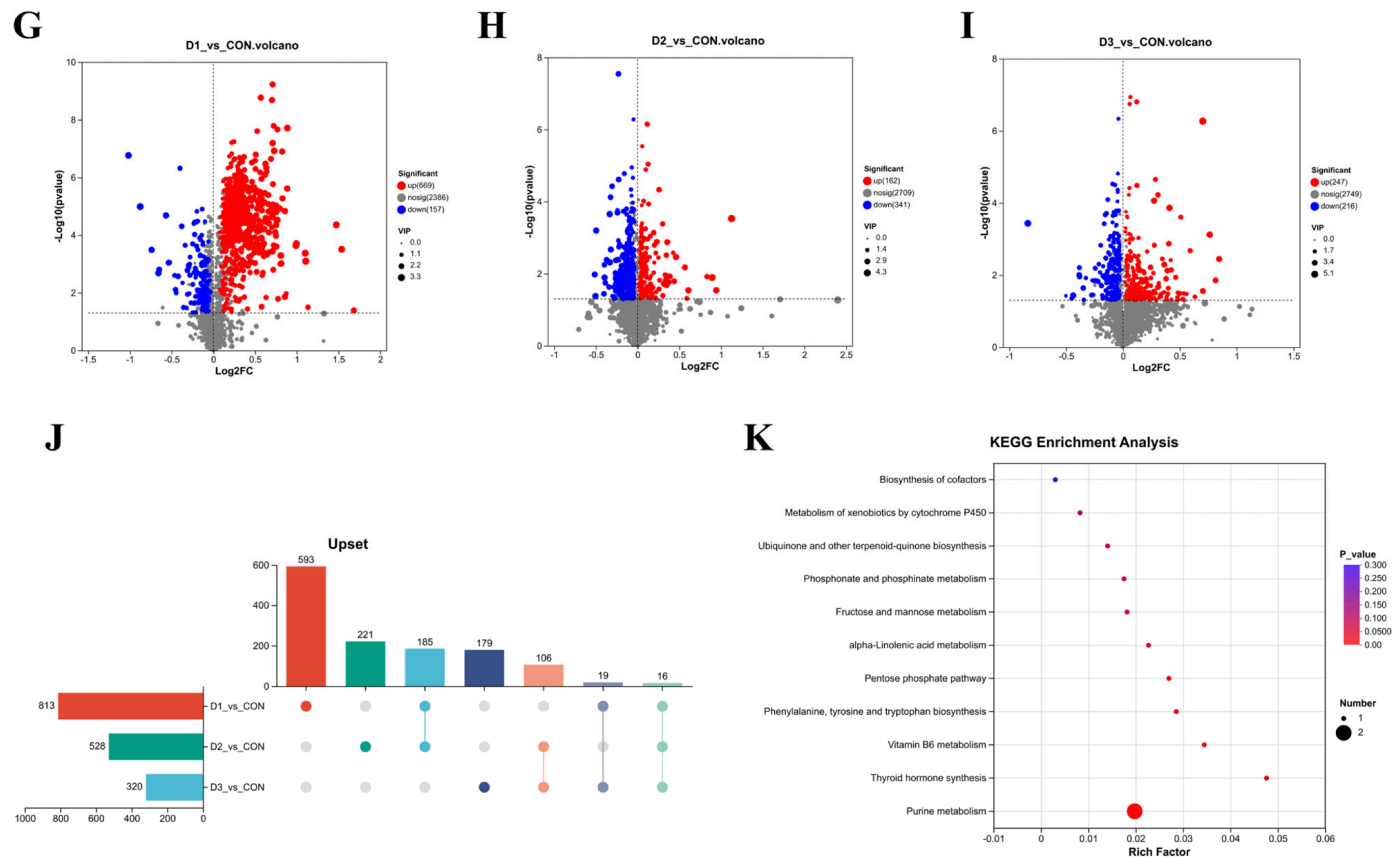
Hepatic metabolomic responses of mice receiving dietary DHM. (**A**–**C**) Positive-ion mode: PCA, PLS-DA analysis, and PLS-DA permutation test; (**D**–**F**) negative-ion mode: PCA, PLS-DA analysis, and PLS-DA permutation test; (**G**–**I**) volcano plots of differential metabolites. The vertical dashed line indicates Log2FC = 0, and the horizontal dashed line indicates the statistical significance threshold (*p* = 0.05); (**J**) UpSet plot of shared and specific differential metabolites; and (**K**) KEGG pathway enrichment bubble plot of shared differential metabolites. CON, basal diet; D1, D2, and D3 refer to mice receiving the basal diet plus DHM at 50, 100, and 200 mg/kg diet, respectively.

Liver tissues were subjected to liquid chromatography–mass spectrometry (LC-MS)-based untargeted metabolomic profiling to examine DHM-related changes in hepatic metabolism. Principal component analysis (PCA) showed partial separation between CON and the DHM-supplemented groups, indicating that DHM may affect the hepatic metabolome (Figure 10A,D). Partial least squares discriminant analysis (PLS-DA) was then applied to further evaluate differences in metabolic profiles among groups. The PLS-DA plots showed clearer separation than PCA, although a small overlap was still present among the DHM-treated groups (Figure 10B,E). These results suggest that dietary DHM supplementation was associated with changes in hepatic metabolic profiles. Permutation testing showed no obvious overfitting of the PLS-DA model (Figure 10C,F).

#### 3.8.2. Differential Metabolite Analysis

To further evaluate DHM-related changes in hepatic metabolites, pairwise comparisons were conducted between each DHM-supplemented group and CON. Differential metabolites were identified using statistical screening and displayed in volcano plots. In the D1 vs. CON comparison, 669 metabolites were upregulated and 157 were downregulated (Figure 10G). For D2 vs. CON, 162 metabolites showed increased abundance, whereas 341 metabolites showed decreased abundance (Figure 10H). In the D3 vs. CON comparison, 247 metabolites were elevated and 216 metabolites were reduced (Figure 10I). In the volcano plots, red points represent upregulated metabolites, and blue points represent downregulated metabolites.

#### 3.8.3. Metabolic Pathway Analysis

The three comparison sets, D1_vs_CON, D2_vs_CON, and D3_vs_CON, were analyzed using Venn analysis and visualized using an UpSet plot to show the number and intersections of differential metabolites identified in the treatment groups compared with the CON group (Figure 10J). Considering the three pairwise comparisons together, 1661 metabolites met the criteria for differential abundance. Overlap analysis of the three metabolite sets retained 16 shared metabolites that were potentially responsive to DHM supplementation.

To further interpret the metabolic relevance of these shared metabolites, the 16 common differential metabolites were mapped to the Kyoto Encyclopedia of Genes and Genomes (KEGG) database for pathway enrichment analysis. As presented in Figure 10K, these DHM-related metabolites were mainly associated with purine metabolism, thyroid hormone synthesis, vitamin B6 metabolism, phenylalanine, tyrosine and tryptophan biosynthesis, the pentose phosphate pathway, and alpha-linolenic acid metabolism. Together, these enriched pathways suggested that dietary DHM supplementation was associated with broad changes in hepatic metabolic profiles.

### 3.9. Hcy Metabolism-Related Parameters

**Figure 11:**
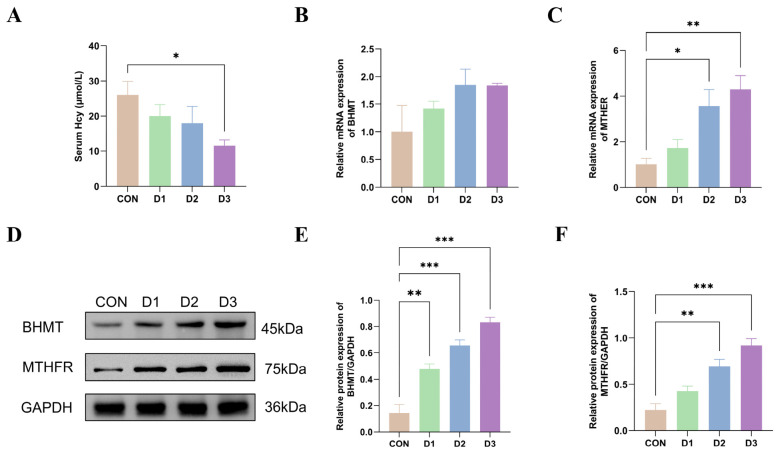
Effects of dietary DHM supplementation on Hcy metabolism-related parameters in mice. (**A**) Serum homocysteine (Hcy) concentration; (**B**) relative mRNA expression of betaine-homocysteine methyltransferase (BHMT); (**C**) relative mRNA expression of methylenetetrahydrofolate reductase (MTHFR); (**D**) Western blot analysis of BHMT and MTHFR protein expression; (**E**) BHMT protein band intensity; and (**F**) MTHFR protein band intensity. All data are presented as the mean ± standard error of the mean (SEM). Significant differences are indicated by “*”, “**”, and “***” (* *p* < 0.05, ** *p* < 0.01, and *** *p* < 0.001); CON, basal diet; D1, D2, and D3 refer to mice receiving the basal diet plus DHM at 50, 100, and 200 mg/kg diet, respectively.

To evaluate whether DHM affects Hcy metabolism-related parameters, we measured serum Hcy levels and hepatic BHMT and MTHFR expression. Both mRNA and protein expression levels were assessed. As shown in Figure 11A, DHM treatment decreased serum homocysteine levels compared with the CON group. This decrease was significant in the D3 group (*p* < 0.05). Hepatic BHMT mRNA expression tended to increase after DHM treatment (Figure 11B). Hepatic MTHFR mRNA expression was increased in the D2 group (*p* < 0.05) and D3 group (*p* < 0.01). Western blot analysis showed that DHM treatment significantly increased hepatic BHMT protein expression (*p* < 0.001). Hepatic MTHFR protein abundance was also markedly elevated (*p* < 0.001; Figure 11D–F).

## 4. Discussion

DHM is a plant-derived flavonoid with antioxidant and metabolic regulatory activities [19]. However, the nutritional role of DHM as a diet-derived bioactive compound in maintaining homeostasis during basal physiological states has not been well characterized. Here, DHM inclusion in the diet strengthened antioxidant-related responses and supported jejunal barrier-associated features, along with shifts in the cecal microbiota, liver metabolic profiles, and Hcy-linked markers. Together, these results offer an integrated view of DHM and point to its potential relevance for redox balance, intestinal barrier integrity, and hepatic metabolic homeostasis.

### 4.1. DHM Supported Growth-Related Traits and Serum Biochemical Status in Mice

Growth-related traits and serum biochemical profiles provide useful information for assessing the dietary application potential of DHM. In this study, dietary DHM supplementation increased 0–21 d BWG in mice, with a significant effect in the D2 group (*p* < 0.05). Several researchers used enterotoxigenic *Escherichia coli* (ETEC)-challenged weaned piglets and found that dietary dihydromyricetin improved their growth performance [20]. Although that study was conducted under an ETEC-challenge condition, it partly supports the possibility that dietary DHM may benefit growth-related traits under specific physiological or stress-related contexts.

Serum biochemical analysis showed that AST decreased (*p* < 0.05), whereas ALT, ALP, TBil, Crea, and Urea remained stable. In addition, TG levels were reduced (*p* < 0.01), HDL-C levels were increased (*p* < 0.05), and TChol and LDL-C levels remained unchanged. Because the present study was conducted in healthy mice fed a normocaloric basal diet, the decrease in AST and the increase in HDL-C were interpreted as moderate biochemical shifts under basal physiological conditions rather than as disease-corrective effects. The decrease in AST, together with unchanged ALT, ALP, TBil, Crea, and Urea levels, suggests that dietary DHM supplementation did not induce obvious abnormalities in serum indicators related to hepatic and renal status [21,22]. The reduction in TG and elevation of HDL-C, together with unchanged TChol and LDL-C levels, indicate a favorable but moderate lipid metabolism-related shift [23,24]. A meta-analysis of randomized controlled trials reported that green tea intake reduced fasting serum total cholesterol and LDL-C concentrations in adults, supporting the potential of tea polyphenol-related dietary interventions to moderately modulate serum lipid profiles [25]. The liver H&E results further supported this interpretation, as no obvious hepatic histopathological abnormalities were observed.

Overall, the serum biochemical and hepatic histological findings suggest that dietary DHM supplementation was not associated with adverse hepatic or renal responses at the tested doses, while the changes in TG and HDL-C indicate a favorable lipid metabolism-related shift.

### 4.2. DHM Improved Hepatic Redox–Inflammatory Status, Potentially Involving Nrf2/HO-1-Related Antioxidant Defense

Oxidative stress is an important factor affecting animal health, nutrient utilization, intestinal barrier integrity, and hepatic metabolic homeostasis. Excessive reactive oxygen species production or insufficient antioxidant defense can induce lipid peroxidation, cellular structural damage, and metabolic dysfunction [26]. MDA reflects the degree of lipid peroxidation. Elevated MDA concentrations usually indicate increased oxidative injury. SOD, T-AOC, GSH-Px, and CAT are commonly measured to evaluate antioxidant defense status. In this research, dietary DHM lowered serum and hepatic MDA concentrations and enhanced T-AOC and GSH-Px activity in mice. These results indicate that DHM reduced lipid peroxidation and enhanced systemic and hepatic antioxidant capacity.

Nrf2 is a key regulator of cellular redox defense and can modulate the transcription of genes related to antioxidant enzymes and detoxification [27]. As a downstream antioxidant protein regulated by Nrf2, HO-1 is widely used as a marker of Nrf2-mediated antioxidant defense [28]. Previous evidence suggests that the Nrf2/HO-1 axis is an important molecular target for natural bioactive compounds to reduce oxidative stress [29,30,31]. DHM has also been reported to reduce cellular oxidative damage through Nrf2/HO-1-related signaling [32]. In this study, Western blotting further revealed that dietary DHM supplementation elevated Nrf2 protein abundance in the liver. Nrf2 protein expression was significantly increased in the D1 and D2 groups (*p* < 0.05) and markedly increased in the D3 group (*p* < 0.01). HO-1 protein expression also showed an upward trend after DHM treatment, with a significant increase in the D3 group (*p* < 0.05). These results were consistent with the decrease in MDA and the increases in T-AOC and GSH-Px. Thus, the DHM-related improvement in antioxidant capacity may partly involve hepatic Nrf2/HO-1-associated antioxidant defense. Similar evidence has been reported in previous DHM studies in animals. Wei et al. [12] reported that DHM enhanced intestinal antioxidant capacity in growing-finishing pigs, with involvement of ERK/Nrf2/HO-1 signaling. Zhu et al. [8] also showed that DHM protected IEC-6 cells and mouse intestine from AFB1-related damage and attenuated oxidative stress through Nrf2/HO-1 pathway activation.

It is worth noting that DHM significantly upregulated hepatic Gclc mRNA expression in mice (*p* < 0.05). Gclc encodes the catalytic component of glutamate–cysteine ligase (GCL). This enzyme controls the rate-limiting step of glutathione (GSH) synthesis. Therefore, Gclc plays an essential role in GSH biosynthesis [33]. Nrf2 can regulate GCL/Gclc transcription and thereby affect GSH-related antioxidant defense [34]. McWalter et al. [35] demonstrated that isothiocyanates induce antioxidant genes such as GCL through an Nrf2-dependent mechanism. Yang et al. [36] also demonstrated that Nrf1 and Nrf2 can regulate GCLC promoter-related transcriptional activity. The GSH system contributes to cellular redox balance, while GSH-Px relies on GSH to eliminate peroxides [37]. Thus, the significant upregulation of Gclc mRNA provides transcriptional support for the enhancement of glutathione-related antioxidant defense by DHM. In this study, the upregulation of Gclc expression was consistent with the increases in serum and hepatic GSH-Px and T-AOC and the decrease in MDA. This further suggests that DHM may improve redox status in mice by supporting Nrf2/HO-1-related antioxidant defense and glutathione-related antioxidant responses.

Nrf2 activity is primarily governed by the Keap1–Nrf2–ARE signaling axis at the molecular level [38]. Under physiological conditions, Keap1 sequesters Nrf2 in the cytoplasm and facilitates its ubiquitination followed by proteasomal degradation. When cells encounter oxidative or electrophilic challenges, this Keap1-dependent repression is attenuated. As a result, Nrf2 becomes more stable and can participate in the transcriptional control of antioxidant and cytoprotective genes. After entering the nucleus, Nrf2 interacts with small Maf proteins and recognizes antioxidant response elements, leading to the induction of downstream targets such as HO-1 and genes associated with glutathione biosynthesis [39]. In the present study, dietary DHM supplementation increased hepatic Nrf2 and HO-1 protein abundance and upregulated Gclc mRNA expression. These molecular changes were in line with the improved hepatic antioxidant status, including higher T-AOC and GSH-Px activity and lower MDA concentrations.

Oxidative stress is also closely linked to inflammatory regulation. Although ROS participate in physiological signaling, excessive ROS production can disrupt redox homeostasis and facilitate inflammatory responses [40,41]. In this study, the improved hepatic redox status after DHM supplementation was accompanied by a significant decrease in TNF-α concentration and a significant increase in IL-10 concentration, while IL-1β and IL-6 showed decreasing trends. These results suggest a shift toward a less pro-inflammatory hepatic cytokine profile. Previous studies have also indicated that Nrf2 may influence inflammatory responses by regulating pro-inflammatory cytokine transcription [42]. Therefore, dietary DHM supplementation may contribute to the maintenance of hepatic redox and inflammatory homeostasis under basal physiological conditions, potentially through Nrf2/HO-1-related antioxidant defense and glutathione-associated responses.

It should be noted that Western blotting was performed using total hepatic protein lysates rather than separately isolated nuclear and cytoplasmic fractions. Therefore, the increased Nrf2 protein abundance observed in the present study reflects changes in total hepatic Nrf2 levels but does not directly demonstrate nuclear translocation or DNA-binding activity. Nevertheless, the concomitant increases in HO-1 protein abundance and Gclc mRNA expression support the involvement of an Nrf2-related antioxidant response.

### 4.3. DHM Improved Jejunal Morphology and Barrier Function

The jejunum is an important part of the small intestine. It also plays a key role in nutrient digestion and uptake. An intact mucosal structure is closely related to efficient intestinal absorption and barrier function. An increase in villus height usually reflects expansion of the intestinal absorptive surface. The villus height-to-crypt depth ratio is also an important morphological indicator, and higher values are generally linked to stronger digestive and absorptive capacity and better mucosal structural stability. Previous animal studies also showed that increased villus height and villus-to-crypt ratio can reflect improved intestinal digestive and absorptive function [43]. In this study, H&E staining showed that DHM supplementation improved jejunal morphology. Jejunal villus height was significantly higher in the D2 group (*p* < 0.05). The lower crypt depth and higher villus height-to-crypt depth ratio further suggest improved jejunal mucosal architecture and absorptive potential after DHM supplementation.

The intestinal barrier protects the host from harmful luminal factors and is essential for maintaining intestinal homeostasis. Structurally, it is formed by the columnar epithelial layer and tight junctions between adjacent epithelial cells [44]. Occludin and ZO-1 are key components of the tight junction complex [45,46]. In this study, immunohistochemistry and Western blotting showed that DHM treatment increased jejunal Occludin and ZO-1 protein expression. Previous DHM-related studies also showed that DHM improved intestinal barrier injury and inflammatory status in models of high-fat diet, DSS-induced colitis, and exercise stress [47,48,49]. These studies and our results suggest that DHM may help strengthen jejunal epithelial tight junction structure and barrier integrity.

Intestinal permeability is strongly influenced by the integrity of the epithelial barrier, as intestinal epithelial cells and tight junction complexes control the movement of luminal microbial products into the circulation [50]. In the present study, dietary DHM supplementation improved jejunal morphology and upregulated Occludin and ZO-1, two tight junction-associated proteins that play important roles in epithelial barrier regulation. To further link these barrier-related changes with microbial product translocation, serum LPS concentration was assessed as a supplementary marker of systemic exposure to gut-derived endotoxin. LPS is a microbial endotoxin derived mainly from the outer membrane of Gram-negative bacteria, and elevated circulating LPS levels are generally associated with barrier disruption and increased translocation of microbial products [51,52]. In this study, serum LPS concentration was reduced after dietary DHM supplementation (*p* < 0.05). Together with the increased expression of Occludin and ZO-1, this result provides additional support for the possibility that dietary DHM may contribute to intestinal barrier-related homeostasis and reduce systemic exposure to gut-derived endotoxin in healthy mice.

### 4.4. DHM Modulated Cecal Microbiota Composition

Cecal microbiota was also clearly reshaped along with the improvement in jejunal structure. Gut microbiota is an important link between dietary nutrition and host intestinal health, nutrient utilization, and metabolic homeostasis. Dietary components can regulate gut microbial composition and affect host physiological functions through microbial fermentation products [53,54]. In this study, dietary DHM supplementation increased microbial richness and diversity and reshaped the overall community profile.

DHM significantly increased the relative abundance of taxa related to Ligilactobacillus, [Eubacterium]_coprostanoligenes_group, Clostridia_UCG-014, NK4A214_group, Prevotellaceae, Family_XIII_AD3011_group, and Clostridia_vadinBB60_group. Ligilactobacillus belongs to lactic acid bacteria-related taxa, and previous evidence suggests that these bacteria may strengthen intestinal epithelial tight junctions and barrier function [55]. Clostridia- and Lachnospiraceae-related taxa are often associated with anaerobic fermentation and short-chain fatty acid production [56,57]. Prevotellaceae-related taxa usually have a strong capacity to utilize complex carbohydrates [58]. In this context, SCFAs may represent important microbial metabolites linking DHM-associated microbiota remodeling with host intestinal and metabolic responses [59]. Acetate, propionate, and butyrate are the predominant SCFAs produced by gut microbial fermentation [60]. Among them, butyrate can serve as an energy source for intestinal epithelial cells and is closely associated with epithelial barrier maintenance, tight junction integrity, and mucosal immune regulation [58]. Propionate and acetate may also participate in gut–liver communication and host metabolic regulation through receptor-mediated signaling [61]. Therefore, the enrichment of anaerobic fermentation-related taxa and complex carbohydrate-utilizing taxa observed in the present study may indicate a shift in SCFA-related fermentative potential. Taxa related to [Eubacterium]_coprostanoligenes_group may participate in the conversion of intestinal cholesterol to coprostanol and may be related to cholesterol excretion and lipid metabolism regulation [62]. These changes were consistent with the improved jejunal morphology, increased Occludin and ZO-1 expression, enhanced antioxidant capacity, decreased TG levels, and increased HDL-C levels observed in this study.

Overall, dietary DHM supplementation increased cecal microbial richness and diversity in mice and promoted the enrichment of some lactic acid bacteria, anaerobic fermentation-related taxa, and lipid metabolism-related taxa. These findings suggest that DHM-associated changes in the cecal microbiota, potentially involving SCFA-related microbial metabolic activity, may be linked to intestinal barrier-related phenotypes and nutritional metabolic homeostasis.

### 4.5. DHM Regulated Hepatic Metabolic Profiles and Was Accompanied by Changes in Hcy Metabolism-Related Parameters

Hepatic untargeted metabolomics further showed that DHM affected multiple nutrition-related metabolic networks. The liver plays a central role in nutrient transformation, lipid use, amino acid turnover, and redox balance in animals. In the present study, DHM reshaped hepatic metabolic profiles and was linked to several enriched metabolic pathways. KEGG analysis indicated that the shared differential metabolites were mainly associated with purine metabolism, vitamin B6 metabolism, phenylalanine, tyrosine and tryptophan biosynthesis, the pentose phosphate pathway, alpha-linolenic acid metabolism, and other metabolism-related pathways. These findings suggest that DHM may affect hepatic metabolism through coordinated changes in energy metabolism, amino acid conversion, lipid utilization, and redox regulation.

Purine metabolism contributes to nucleotide formation and energy supply, while the pentose phosphate pathway supports NADPH generation and cellular reducing capacity. Purine nucleotides are the basis for DNA and RNA synthesis and also participate in energy transfer and cellular signaling through ATP and GTP [63]. The pentose phosphate pathway provides ribose-5-phosphate for nucleotide synthesis and produces NADPH to support reducing power and antioxidant defense [64]. Thus, changes in purine metabolism and the pentose phosphate pathway may reflect the combined effects of DHM on hepatic energy supply, nucleotide metabolism, and antioxidant adaptation.

Notably, the differential metabolites were also enriched in the vitamin B6 metabolism pathway. Vitamin B6 is an important cofactor for many amino acid-metabolizing enzymes and participates in the transsulfuration process, which converts Hcy into cysteine. Cysteine is an important precursor for GSH synthesis, and GSH is a key substrate for GSH-Px to remove peroxides and maintain cellular redox homeostasis. Thus, vitamin B6 metabolism may link Hcy metabolism, one-carbon metabolism-related networks, and the GSH-dependent antioxidant system. Previous studies have shown that the vitamin B6-dependent transsulfuration pathway links one-carbon metabolism with the glutathione peroxidase system [65]. Together with the increased hepatic T-AOC and GSH-Px activity and reduced MDA levels, these findings suggest that DHM-induced changes in vitamin B6-related metabolism may help enhance hepatic antioxidant defense.

In addition, alpha-linolenic acid metabolism is closely related to fatty acid composition, lipid utilization, and inflammatory regulation [66]. In this study, DHM decreased serum TG levels and increased HDL-C levels, suggesting that DHM may help improve lipid metabolic status in mice. Together with changes in the alpha-linolenic acid metabolism pathway, this result suggests that DHM may help maintain hepatic lipid metabolic homeostasis by regulating fatty acid metabolism and lipid transport-related processes. Consistent with our findings, Guo et al. observed that dietary DHM supplementation improved lipid metabolic status and antioxidant responses in finishing pigs [67].

Alongside global metabolic profile changes, this study also observed changes in Hcy metabolism-related parameters. Hcy occupies a central position in the methionine cycle and one-carbon metabolic network, with remethylation and transsulfuration serving as its main metabolic routes [68]. BHMT participates in betaine-dependent Hcy remethylation and catalyzes the conversion of Hcy back to methionine. MTHFR catalyzes the conversion of 5,10-methylenetetrahydrofolate to 5-methyltetrahydrofolate and provides the methyl donor for folate-dependent Hcy remethylation [69,70,71]. In this study, DHM decreased serum Hcy levels and increased hepatic BHMT and MTHFR expression, suggesting that DHM may be associated with enhanced Hcy remethylation-related processes and may help maintain Hcy metabolic homeostasis.

Taken together, the hepatic untargeted metabolomic results and Hcy-related measurements suggest that dietary DHM supplementation was associated with coordinated changes in nutrient-related hepatic metabolic networks, including nucleotide turnover, reducing-power generation, fatty acid metabolism, and Hcy remethylation-related processes. However, these findings should be interpreted as metabolic associations under basal dietary conditions rather than direct evidence of metabolic protection. In addition to the lack of quantitative measurements of SAM, SAH, betaine, folate, and other representative one-carbon metabolites, dynamic glucose homeostasis was not evaluated using glucose tolerance testing; therefore, the effects of dietary DHM supplementation on systemic glucose tolerance remain to be determined, particularly under metabolic-stress conditions. Further studies using metabolic challenge models would be helpful. For example, a high-fat diet-induced overnutrition model could be used to examine whether DHM can attenuate diet-induced hepatic lipid accumulation, oxidative stress, inflammatory responses, gut barrier impairment, glucose tolerance disturbances, and gut–liver axis dysregulation.

## 5. Conclusions

Overall, dietary DHM supplementation supported hepatic redox–inflammatory homeostasis in mice under basal physiological conditions, as reflected by improved antioxidant status and a less pro-inflammatory hepatic cytokine profile. This hepatic response was accompanied by improvements in jejunal barrier-related phenotypes and broader changes in cecal microbiota composition, hepatic metabolomic profiles, and Hcy metabolism-related markers. These results indicate that DHM may contribute to coordinated nutritional adaptation across hepatic antioxidant defense, inflammatory regulation, intestinal barrier function, microbial ecology, and hepatic metabolic homeostasis. This study provides a multi-level nutritional profile of DHM and supports its potential relevance as a plant-derived dietary bioactive compound for functional food development. Future studies incorporating Nrf2 functional validation, targeted metabolomics, and metagenomics are required to clarify the causal basis of these responses.

## Figures and Tables

**Table 1:** List of primers used for qRT-PCR analysis.

Gene	Direction	Primer Sequence (5′–3′)
GAPDH	Forward	GCACCGTCAAGGCTGAGAAC
	Reverse	TGGTGAAGACGCCAGTGGA
ZO-1	Forward	CTCACAGTACAGCCAGCCAG
	Reverse	GGTGGGTCTGGTTTGGACAT
Occludin	Forward	AAGTGAATGGCAAGCGATCATA
	Reverse	CTGTACCGAGGCTGCCTGAA
Nrf2	Forward	TATCTCCTAGTTCTCCGCTGCTC
	Reverse	GTGGCAACTCCAAGTCCATCAT
HO-1	Forward	CTGGAGATGACACCTGAGGTCAA
	Reverse	CTGACGAAGTGACGCCATCTG
Gclc	Forward	CACATCTACCACGCAGTCAAG
	Reverse	ATCGCCTCCATTCAGTAACAAC
MTHFR	Forward	CCCTATCCTGCCTGGGATCTT
	Reverse	TCAATGCCGTAGTTGCGGATG
BHMT	Forward	AGAACGCTTAAATGCCGGAGA
	Reverse	CGATGAAGCTGACGAACTGC

**Table 2:** Growth performance of mice fed diets supplemented with DHM.

Parameters	CON	D1	D2	D3	SEM	*p*-Value
Initial body weight	11.14	11.20	11.18	11.33	0.22	0.941
0–7 d BWG	4.58	4.80	5.21	5.05	0.10	0.128
0–14 d BWG	6.32	6.47	7.18	6.64	0.13	0.084
0–21 d BWG	6.64	6.77	7.50	6.85	0.11	0.045
0–28 d BWG	7.45	7.70	8.34	7.67	0.12	0.057

Note: BWG, body weight gain; SEM, standard error of the mean; CON, basal diet; D1, D2, and D3 refer to mice receiving the basal diet plus DHM at 50, 100, and 200 mg/kg diet, respectively.

## Data Availability

All data generated or analyzed during this study are included in the article and Supplementary Materials. Further inquiries can be directed to the corresponding author.

## Supplementary Materials

The following supporting information can be downloaded at: https://www.mdpi.com/article/10.3390/nu18142390/s1, Table S1: Composition and nutrient levels of the basal diet; Table S2: Liver index of mice fed diets supplemented with DHM; Figure S1: Effects of dietary DHM supplementation on serum LPS concentration in mice.
