# Supplementary material for: Dietary Dihydromyricetin Supplementation Enhances Antioxidant Capacity and Modulates Jejunal Barrier Function, Cecal Microbiota, and Hepatic Metabolism in Mice
Source: Nutrients. 2026 Jul 22;18(14):2390. doi: 10.3390/nu18142390 (PMC13414717; doi:10.3390/nu18142390)
Supplement: Supplementary file 1 [file nutrients-18-02390-s001.zip › nutrients-4384262-supplementary.pdf]

Supplementary Materials

Table S1. Composition and nutrient levels of the basal diet.

Ingredients	Content (%)	Nutrient composition	Content
Corn	48.30	Crude protein (%)	21.13
Wheat middlings	14.00	Crude fat (%)	4.83
Wheat	10.00	Crude fiber (%)	2.54
Alfalfa hay	2.00	Crude ash (%)	5.87
Soybean meal	5.00	Calcium (%)	1.43
Peruvian fish meal	8.30	Total phosphorus (%)	0.84
U.S. chicken meal	6.00	Carbohydrates (%)	56.63
Animal premix	4.30	Calculated energy (kcal/kg)	3545.2
Calcium carbonate	1.10	Protein energy ratio (%)	24
Soybean oil	1.00	Fat energy ratio (%)	12
Total	100.00	Carbohydrate energy ratio (%)	64

Table S2. Liver index of mice fed diets supplemented with DHM.

Parameters	CON	D1	D2	D3	*p*-value
Liver index (%)	4.14 ± 0.09	4.09 ± 0.08	3.88 ± 0.09	4.12 ± 0.07	0.130

Note: Liver index (%) was calculated as liver weight divided by final body weight × 100%. CON, basal diet; D1, D2, and D3 refer to mice receiving the basal diet plus DHM at 50, 100, and 200 mg/kg diet, respectively.

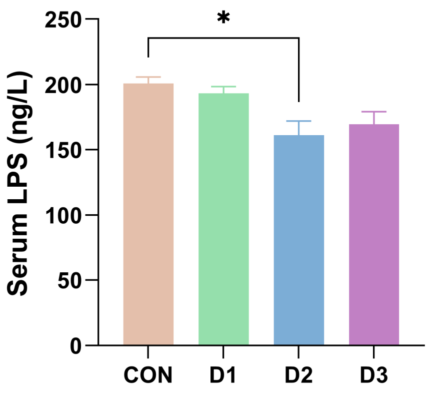

Figure S1. Effects of dietary DHM supplementation on serum LPS concentration in mice. Data are presented as the mean ± standard error of the mean (SEM). Significant differences are indicated by "*" (*$p < 0.05$); CON, basal diet; D1, D2, and D3 refer to mice receiving the basal diet plus DHM at 50, 100, and 200 mg/kg diet, respectively.
